# Supplementary material for: Learning curve and surgical outcome of robotic assisted colorectal surgery with ERAS program
Source: Sci Rep. 2022 Nov 29;12:20566. doi: 10.1038/s41598-022-24665-w (PMC9709162; doi:10.1038/s41598-022-24665-w)
Supplement: Supplementary file 1 — Supplementary Information 1. [file 41598_2022_24665_MOESM1_ESM.zip › LSC Raw data-Ñ[▒K/144/P_ñw╝╨░O▒Kñσ.pdf]

[redacted]

Pathologic diagnosis:
1. Sigmoid colon, laparoscopic anterior resection ---
a. Adenocarcinoma, moderately to poorly differentiated. Tumor invades to visceral peritoneum, and metastasizes to mesocolic lymph nodes (9/12) and peritoneal surface of ovarian adnexal soft tissue.
b. Tubular adenoma with focal high-grade dysplasia.
The resection margins, ovary, and fallopian tube are free of tumor
2. IMA root, dissection --- Unremarkable fibroadipose tissue. No lymphoid tissue seen.
3. Colon, cut end, distal, resection --- Negative for malignancy.

Ancillary study for diagnosis:
IHC stains for PMS2 and MSH6 are done on section N. Both markers are preserved.

Prognostic and predictive factor:
1. Tumor size: 6.4x2 cm.
2. Depth of invasion: Visceral peritoneum (including tumor continuous with serosal surface through area of inflammation).
3. Histologic grade: Moderately to poorly differentiated.
4. Angiolymphatic invasion: Present.
5. Perineural invasion: Present.
6. Tumor deposits (discontinuous extramural extension): Present, specify number of deposits: multiple (> 10).
7. Macroscopic tumor perforation: Not identified.
8. Surgical Margins:
a) Proximal Margin: Uninvolved by invasive carcinoma, distance of tumor from margin: 3.8 cm.
b) Distal Margin: Uninvolved by invasive carcinoma, distance of tumor from margin: 2.6 cm.
c) Radial or Mesenteric Margin: Uninvolved by invasive carcinoma, distance of tumor from margin: 5 cm.
9. Lymph node status: Involved (9/12).
10. Extranodal involvement: Present.
11. Tumor regression grading S/P CCRT: No known presurgical therapy.
12. Type of polyp in which invasive carcinoma arose: Not identified.
13. Additional pathologic findings: Tubular adenoma.
14. Pathological TNM Stage: pT4aN2bM1c (According to the eighth edition, American Joint Committee on Cancer Staging Guidelines for Tumors).
15. TNM descriptors: Not applicable.

Gross description:
The specimen consists of 1) two separated segments of colon. The shorter colon segment, 3.2 cm in length, and 4 cm in both cut ends circumference and mesocolic soft tissue. A tan soft pedunculated polypoid lesion, 1.7x1.2x0.6, with a stalk, 0.8 cm in length, is noted over the mucosa of shorter colon segment, and it is 0.8 cm to the nearest cut end. Both cut ends are free of the lesion grossly. The longer colon segment is 12 cm in length with 6 cm in both cut ends circumference and mesocolic soft tissue. A gray tan firm annular elevated and centrally ulcerative lesion, 6.4x2 cm, is noted over the mucosa of longer colon segment, and the lesion is 2.6 cm to the nearest cut end. On cross section, the lesion involves underlying mesocolic soft tissue with 5 cm to the radial margin and less than 1 mm to serosa grossly. There are multiple nodular lesions, up to 0.2x0.2x0.1 cm, noted at the serosal surface. A fallopian tube, 2.2 cm in length, is grossly adhesion to the serosal surface. Both cut ends are free of the lesion grossly. 2) IMA root, a piece of tan yellow soft tissue, 1x1x0.4 cm. 3) distal cut end, a piece of tan mucosa-covered soft tissue, 1.5x1.3x0.4 cm. Representative part for section: A-D) shorter colon segment of specimen 1 [A-B) both cut ends C) radial margin D) polypoid lesion] E-S) longer colon segment of specimen 1 [E-F) both cut ends G) radial margin H) adhesion site J) fallopian tube K-Q) lesion R-S) serosa] T) specimen 2 U) specimen 3 V-X) mesocolic lymph nodes.

Note:
1. The interpretation of MMR panel and recommended studies are as

1. The interpretation of MMR panel and recommended studies are as below: (1) No loss of nuclear expression of MMR proteins: low probability of microsatellite instability-high (MSI-H) . (2) Loss of nuclear expression of MLH1 and PMS2: testing for methylation of the MLH1 promoter and/or mutation of BRAF is indicated (the presence of a BRAF V600E mutation and/or MLH1 methylation suggests that the tumor is sporadic and germline evaluation is probably not indicated; absence of both MLH1 methylation and of BRAF V600E mutation suggests the possibility of Lynch syndrome, and sequencing and/or large deletion/duplication testing of germline MLH1 may be indicated). (3) Loss of nuclear expression of MSH2 and MSH6: high probability of Lynch syndrome (sequencing and/or large deletion/duplication testing of germline MSH2 may be indicated, and, if negative, sequencing and/or large deletion/duplication testing of germline MSH6 may be indicated). (4) Loss of nuclear expression of MSH6 only: high probability of Lynch syndrome (sequencing and/or large deletion/duplication testing of germline MSH6 may be indicated). (5) Loss of nuclear expression of PMS2 only: high probability of Lynch syndrome (sequencing and/or large deletion/duplication testing of germline PMS2 may be indicated). (6) Please note: There are exceptions to the above IHC interpretations. These results should not be considered in isolation, and clinical correlation with genetic counseling is recommended to assess the need for germline testing.
2. This case has been peer reviewed by two doctors.

#T-59300_2 #M-81403_2 3 1105 S0000F
#T-C4560_2 #M-81406_2 2 1105 000000
#T-88900_2 #M-81406_2 2 1140 000000

已電子簽章

敬告：本單僅供醫師參考使用，不具任何申請效力！！
請病患妥為保管，若不慎遺失需自負個人資料外洩責任！
